# Association between Physical Fitness, Physical Activity Level and Sense of Coherence in Swedish Adolescents; An Analysis of Age and Sex Differences

**DOI:** 10.3390/ijerph191912841

**Published:** 2022-10-07

**Authors:** Anna Hafsteinsson Östenberg, Anton Enberg, Haris Pojskic, Barbara Gilic, Damir Sekulic, Marie Alricsson

**Affiliations:** 1 Department of Sport Science, Linnaeus University, 352 95 Kalmar/Växjö, Sweden; 2 Faculty of Kinesiology, University of Zagreb, 10000 Zagreb, Croatia; 3 Faculty of Kinesiology, University of Split, 21000 Split, Croatia

**Keywords:** physical, social capacity, sedentary behavior, screen time, psychological resilience, muscular strength, aerobic capacity

## Abstract

Introduction: The aim of this study was to investigate the association between physical fitness, physical activity, and sense of coherence (SOC) in Swedish adolescents (n = 2028 males, n = 1287 females) aged 14 to 18 using a cross-sectional design. Methods: Using the Swedish Physical power Mental harmony and Social capacity (FMS) student profile, participants performed physical tests measuring their cardiovascular ability (CV) and muscular strength. Questionnaires were used to measure physical activity levels (PA), the participation in organized physical training, sedentary behavior (SB), screen time, and SOC value. Multiple linear regression analyses were used to analyze the association between SOC and independent variables. Results: The regression analyses explained a small, shared variance between SOC and the independent variables in boys (4.3%) and girls (3.3%). SB showed a positive association with SOC both in girls (β = 0.114, *p* = 0.002) and boys (β = 0.109, *p* = 0.013). Abdominal strength was positively associated, while VO_2_max was inversely associated, with SOC (β = 0.113, *p* = 0.022; β = −0.109, *p* = 0.026, respectively) in boys. Girls had poorer fitness than boys did across all age groups except at age 18. PA levels decreased from age 14 to 18 in girls and boys, but without differences between sexes. Abdominal strength decreased from age 14 to 18 in girls and boys. In general, girls had lower SOC than boys across all age groups. Conclusions: Poor sedentary behavior was significantly associated with weaker SOC for both genders, indicating overall physical activity as the most important factor for stronger SOC. However, emotional support in vulnerable environments may have a bigger impact than physical activity or sedentary behavior on the SOC value for adolescents.

## 1. Introduction

Adolescence is an intensive and sensitive period of physical and psychological changes where social relationships and emotional and academic abilities are developed simultaneously [1,2]. It is often described as a period of “storm and stress” [3] due to the onset of many stress-induced mental health problems (e.g., depression, anxiety, and post-traumatic stress) that increase in childhood and peak in adolescence [4,5,6,7]. Mental health problems play a big role in the global burden of disease and affect approximately 10–20% of children and adolescents worldwide [7], while the number of adolescents with emotional and mental problems has increased during the recent decade, particularly in central and northern Europe [8]. One of the major concerns with adolescents’ mental health is that it affects the mental state when they become adults [8,9]. Not surprisingly, the problem of mental health of adolescents is among the most important issues in public health nowadays [10,11]. 

While it is obviously hard to avoid daily stressful situations, developing the ability to cope effectively with stressors is even more important. In the late eighties, Israeli American sociologist Antonovsky developed the concept of a sense of coherence (SOC), trying to explain why some people have better resistance to stress and stay healthy, while others struggle with it and get sick [12]. In general, SOC is a scale that measures how people use their generalized resistance resources (GRR) to deal with stress, as well as maintain and improve their health. Individuals with more available GRR (e.g., personal experience, self-esteem, money, social support, and intelligence) tend to do better coping with stressors and everyday life challenges [13]. SOC assesses three components: meaningfulness, comprehensibility, and manageability of life. Meaningfulness is the motivational component referring to an emotional connection and the worthiness of commitments and engagement in life [14]. Manageability is a behavioral aspect that refers to an individual’s ability to recognize and use available GRR to manage, control, and respond to challenges in life. Comprehensibility is related to the cognitive ability of an individual to perceive and understand the occurring context of both internal and external stimuli that might create stressful and chaotic situations clearly and rationally [15,16]. 

Consequently, it is of great importance to know about factors associated with increased SOC, mental and physical health, and reduced stress factors. For instance, physical activity (PA) showed to have multiple positive effects on physical and mental health. In this regard, increased PA levels showed improved SOC, and positively affected concentration, pleasurable engagement, subjective health perception, and life satisfaction in adolescents [17,18,19]. Furthermore, PA positively contributed to improved self-esteem and self-confidence [20], reduced anxiety, and increased well-being [21,22], while sedentary behavior and insufficient PA were inversely associated with mental health in young people [20,22,23]. Moreover, higher PA was associated with reduced risk of cardiovascular disease and type 2 diabetes [24], lower obesity/overweight [25,26], and improved physical fitness [25,27,28]. 

In general, physical fitness is defined as one’s capacity to perform daily activities and exercises with optimal performance, and it is determined by several components such as cardiorespiratory fitness, muscular strength and endurance, body composition and flexibility [29,30]. It is considered a strong predictor of current and future health in children and adolescents, modulating stress, fatigue, sedentary, and health risk behaviors [2,31]. Unfortunately, it is frequently reported that children and adolescents have an insufficient level of physical fitness [25,32,33], mainly due to decreased PA [23]. What is more, physical fitness and PA levels decline with progressing age. Specifically, a review study that included prospective studies on children and adolescents aged 10–19 years reported an annual decline of 7% in PA levels after age 13 [34].

Additionally, it was reported that the prevalence of PA and cardiovascular fitness level is sex-dependent [25,35,36,37]. Evidently, girls are engaged in PA less than boys [35,36,37]. It was reported that lower PA and physical fitness in girls were associated with their poorer mental health compared to boys. In brief, among 566,829 examined adolescents, girls showed a higher level of anxiety and lower level of satisfaction compared to boys, with the differences being more exaggerated in gender equal countries, such as Sweden and Finland [38]. However, relatively few studies have examined sex-dependent differences in the association between PA, fitness level, and mental health, reporting inconsistent results [39,40]. 

Collectively, it seems reasonable to assume that adolescents with a stronger SOC would mobilize appropriate bodily resources, engaging in behaviors that promote health (e.g., physical activity) and, thus, increase their fitness levels [14,41]. Therefore, the primary aim of the study was to investigate the association between SOC, physical fitness, PA level, screen time, and sedentary behavior separately in Swedish male and female adolescents, according to the Physical power, Mental harmony, and Social capacity (FMS) student profile [26]. A secondary aim was to examine differences between male and female adolescents in PA, fitness level, and sedentary behavior across the age groups. We hypothesized that adolescents with a higher activity level, physical fitness, excessive screen time, and poorer sedentary behavior display a stronger SOC.

## 2. Materials and Methods

### 2.1. Participants

Fourteen- to eighteen-year-old Swedish high-school students (2028 girls; 61% and 1287 boys; 39%) from 45 schools located in three urban regions in the middle and south of Sweden, including communities with smaller (<50,000) and bigger (>50,000) population, were invited and accepted to participate in the study. They were divided into several age groups: girls (age 14, n = 40; age15, n = 239; age 16, n = 1312; age 17, n = 384; and age 18, n = 53) and boys (age 14, n = 40; age 15, n = 137; age 16, n = 773; age 17, n = 260; age 18, n = 77), respectively (Table 1). All participants included in this study met the requirements for inclusion, which were being a Swedish school student, 14–18 years old, attending a school using the FMS instrument, having a complete set of data, e.g., had undergone all the physical tests and answered all the questionnaires included in the FMS instrument during 2014–2020. The FMS profile is a tool designed by the Swedish Institute of Physical, Mental, and Social capacity for adolescents aged 12–19 years old and includes a questionnaire (e.g., PA level and sedentary behavior), fitness tests (e.g., cardiovascular fitness, muscular strength, and flexibility) and anthropometric measurements (e.g., body height and mass) [26]. Parents/guardians and the participants were all informed about the fitness test and the FMS questionnaire. A written consent was signed by the parents/guardians and the students to participate in the study. The present investigation was conducted in accordance with the Declaration of Helsinki for human studies and was approved for retrospective and prospective data by the Swedish Ethical Review Authority (Dnr 2019-05076).

#### Variables and Measurement

The variables in this study included independent variables (age, sex, BMI, upper- and lower-body strength, abdominal strength, VO_2_max, everyday physical activity, organized physical training in a school/club setting, sedentary behavior, and screen time) and a dependent variable (sense of coherence). 

The FMS profile includes anthropometric measurements, questionnaires (including SOC questionnaire), and the fitness tests follow the standardized protocols [26]. Anthropometrics included body mass and height measured by the school nurses. Body mass index (BMI) was calculated for each student [body mass (kg)/body height squared (m^2^)]. Physical education (PE) teachers conducted the fitness tests during the PE sessions.

### 2.2. FMS Questionnaire 

Four single-item questions from the FMS student profile questionnaire were used to measure the prevalence of physical training, everyday PA, sedentary behavior style, and screen time among the participants. (1) Physical training: How often (times a week) do you train in a club, so that you become at least 30 min breathless (e.g., running, playing ball sports, etc.)? (answers included: never, once a week, two times, three times, at least four times). (2) Physical activity: How often (days a week) do you walk, exercise, cycle, or exercise otherwise for at least 30 min in a row? (answers included: (<3 days, 3 days, 4 days, 5 days, >5 days). (3) Sedentary behavior: How many hours of your school and leisure time do you sit still? (answers included: >11 h, 10–11 h, 8–9 h, 6–7 h, <6 h). (4) Screen time: How much time (number of hours) do you spend in front of a screen (e.g., computer, television, mobile phone) during your free time? (answers included: >4 h, 2–4 h, 1–2 h, 0.5–1 h, <0.5 h). The self-reported questionnaires consisted of a Likert-type scale ranging from 1 to 5, and it has previously been validated in large sample studies [26,42,43]. Sedentary behavior and screen time were inversely coded with a higher score, meaning lower sedentary behavior and less screen time. 

### 2.3. Sense of Coherence Questionnaire 

The present study used the shortened 13-item SOC questionnaire. Every question option was ranked 1–7. The item response in the questionnaire was counted as 1 for the lowest rank and 7 for the highest rank. Four questions were counted in a reverse score setting in the 13-item questionnaire. An example of a reverse counted question is “Have people you trust made you disappointed?”; ranking 1 means it “never happens”, and ranking 7 means it “happens a lot.” A high total score in the questionnaire represents a high value of SOC [44]. The dependent SOC variable in the data analysis consisted of the total score of the questionnaire (ranged 13–91). The 13-item questionnaire has shown to be a reliable [44,45,46] and valid [47] instrument, measuring how individuals manage stressful situations and stay well [15]. 

### 2.4. Fitness Measurements

Muscular strength was assessed using the following tests: sit-ups (number of repetitions), upper body strength (arm lift with 5 kg for females and 5 or 10 kg for males (number of repetitions), and lower body strength (static wall squat, i.e., holding position “sitting chair” for as long as possible, measured in seconds). All tests are described elsewhere in more detail [26].

### 2.5. Cardiovascular Fitness

To test cardiovascular fitness, PE teachers could choose one of several maximal and submaximal tests, depending on the available resources, equipment, and the pupils’ characteristics (e.g., overall fitness level, familiarity with the tests, etc.). A Cooper test was most frequently used. It estimates the participant’s maximal oxygen consumption based on the distance covered in 12 min of running [48]. It has shown to be a valid and reliable test for estimating VO_2_max [49]. Alternatives to the Cooper test were the 1-Mile Walk test [50], Step test [51], and Åstrand cycle ergometer test [51,52], all of which were considered valid and reliable. 

### 2.6. Statistical Analyses

The Shapiro–Wilk test was used to analyze the normality of the distribution for all variables. Continuous variables were presented by mean and standard deviation (SD), while one-way analysis of variance (ANOVA) with consecutive Bonferroni post hoc test and the independent *t*-test were used to analyze the differences across the age groups and differences between girls and boys, respectively. For ordinal variables, the Kruskal–Wallis Test and Mann–Whitney Test were used. Standard multiple linear regression analyses were used to analyze the association between the dependent variable (i.e., SOC value) and the independent variables separately for girls and boys. Independent variables consisted of ordinal (i.e., everyday physical activity, physical training, sedentary behavior, and screen time) and continuous variables: anthropometry (i.e., BMI), physical fitness (i.e., VO_2_max, abdominal strength, upper body strength, and lower body strength), and age. Before the calculation of multiple regression, the collinearity between the predictors was checked. The variance inflation factor (VIF) for all predictors was below 2.5, showing low multicollinearity. Therefore, all predictors were included in the multiple regression calculations. Significance level was set at *p* < 0.05. Statistical analyses were performed using IBM SPSS Statistics 27 (IBM, Armonk, New York, NY, USA).

## 3. Results

Descriptive statistics (mean ± SD) and differences across age and sex groups in anthropometric, physical fitness, and SOC variables are presented in Table 1. Differences in physical training frequency, PA level, sedentary behavior, and screen time across age and sex groups are presented in Table 2.

### 3.1. Anthropometrics 

Boys were significantly taller (*p* < 0.005) and heavier (*p* < 0.005) than girls across all age groups (14–18 years old). Body mass increased across all age groups in boys (*f* = 6.07, *p* = 0.001). In contrast, there were significant differences between age groups (*f* = 4.67, *p* = 0.001) in girls but without significant increase across the groups. There were no differences in body height across age groups, either in boys or in girls (*f* = 1.23, *p* = 0.30, *f* = 1.78, *p* = 0.13, respectively). BMI showed no significant differences across age and gender groups. However, girls had significantly higher BMI in ages 16, 17, and 18 compared with age 14. 

### 3.2. Physical Fitness

Boys had higher VO_2_max than girls across all age groups. There was a difference in both girls (*f* = 12.85, *p* = 0.00) and boys (*f* = 9.85, *p* = 0.001) in each age group, with a tendency to decrease with age from 57.3 ± 8.9 mL/kg/min at age 14 to 48.6 ± 13.1 mL/kg/min at age 18 in boys and from 51.1 ± 5.3 mL/kg/min at age 14 to 44.4 ± 7.2 mL/kg/min at age 18 in girls.

Lower-body strength was higher in boys than girls in age groups 16 and 17, with a tendency to decrease with age in both groups (*f* = 2.92, *p* = 0.02; *f* = 4.38, *p* = 0.00, respectively). On the other hand, upper-body strength was better in boys at age 14, but poorer at age 18. Boys had higher abdominal strength (i.e., sit-ups) than girls in all age groups except at age 14. Moreover, it declined with age, both in girls and boys (*f* = 4.61, *p* = 0.00; *f* = 3.90, *p* = 0.00, respectively). 

### 3.3. Sense of Coherence 

Boys had a stronger SOC than girls in age groups 15, 16, and 17. There were no differences in the SOC level between age groups in girls (*f* = 0.38, *p* = 0.82). On the other hand, boys in age group 15 had stronger SOC than those from age group 18. 

### 3.4. Physical Training, Everyday PA, Sedentary Behavior, and Screen Time

Boys had a significantly higher prevalence of physical training in age group 15 (*Z* = −3.115, N1 = 226, N2 = 126, *p* = 0.002) and 16 (*Z* = −4.488, N1 = 1289, N2 = 759, *p* ≤ 0.001), compared to girls. Specifically, 47.7% of boys and 32.7% of girls reported that they trained in sports at a moderate to high-intensity level at least 4 times per week. There was not significant difference between boys and girls in everyday PA. a Significant difference across age groups was found in everyday PA for both girls χ^2^ (4, N = 1973) = 15.427, *p* = 0.004 and boys χ^2^ (4, N = 1245) = 14.914, *p* = 0.005. Prevalence of PA decreased from age group 14 compared to age group 18 for both sexes. Girls had a significantly poorer sedentary behavior compared to boys in age group 16 (Z = −3.335, N1 = 880, N2 = 572, *p* ≤ 0.001), and boys spent significantly more screen time than girls in age group 14 (Z = −3.147, N1 = 40, N2 = 40, *p* = 0.002). There were no significant differences in sedentary behavior and screen time across age groups in any of the sexes.

### 3.5. Factors Associated with a Sense of Coherence 

The multiple regression analyses showed a significant association of the independent variables with SOC both in girls (*p* = 0.003) and boys (*p* = 0.005) (Table 3). However, the used predictors explained only 3.3% (R^2^ = 0.033) variability of SOC in girls and 4.3% (R^2^ = 0.043) in boys. Sedentary behavior showed a significant positive association with SOC both in girls (β = 0.114, *p* = 0.002) and boys (β = 0.109, *p* = 0.013), meaning the better sedentary behavior, the stronger SOC. Abdominal strength was positively associated, while VO_2_max was inversely associated with SOC (β = 0.113, *p* = 0.022; β = −0.109, *p* = 0.026, respectively), in boys. There were no other significant associations between independent variables and SOC. 

## 4. Discussion

The main finding in the present study was that adolescents with less sedentary behavior had stronger SOC. Moreover, abdominal strength showed positive association, while VO_2_max had a negative association with SOC in boys. They also had a higher value of SOC and VO_2_max across all age groups compared to girls. Differences between girls and boys in everyday physical activity, sedentary lifestyle, and screen time were small. However, there was a significantly higher prevalence of physical training frequency for boys compared to girls in age groups 15–16. 

### 4.1. Determinants of Sense of Coherence (SOC)

The hypothesis that adolescents with a higher PA level, physical fitness, and poorer sedentary behavior would display a stronger SOC irrespective of gender was partially supported. The present study showed less sedentary behavior being associated with a stronger SOC in adolescents, irrespective of the sex and age. This is consistent with a recent study showing a weak SOC being associated with insufficient PA [53]. Moreover, the finding is in line with several studies showing higher sedentary behavior being associated with worse mental health [20,22,54,55]. Furthermore, Iannotti et al. (2009) suggest the sedentary behavior and mental health may be affected by time spent watching television, which, in turn, reduces social interaction, personal problem-solving situations, and does not activate and challenge one’s cognitive and physical capacities [56]. It seems that screen time and usage of social media increase not only sedentary time but also lead to social pressure and body dissatisfaction among adolescents [57,58]. However, the social media and screen time can provide some sort of social connection and interaction among adolescents, which could explain the non-significant association between screen time and SOC in the present study. 

Unexpectedly, there were no significant associations between SOC and physical training (PT) and everyday PA, which is in line with some previous studies [13,19], but opposing to others that showed a positive association between PA and mental health [59,60]. The use of a single item (e.g., question) to assess adolescent PA and PT might be a possible explanation for the lack of association between PA, PT, and SOC.

Furthermore, BMI was not significantly associated with SOC. The present findings support previous studies which have suggested that BMI and mental health are not associated with adolescents younger than 20 years [61], or when controlling for fitness variables [62,63]. 

The only strength variable positively associated with SOC was boys’ abdominal strength that was tested in the same way across all age groups and both sexes. The result is in line to previous research where strength is positively associated with mental health outcomes, such as self-esteem and self-perceptions [64], physical self-perception [65], perceived health status and life satisfaction [66], and lower depression [67]. However, in the current study there were no observed association between strength of other muscle groups (e.g., upper- and lower-body) and SOC. Possible explanation can be that boys aged 14–16 had the option of choosing either a 5 kg or a 10 kg dumbbell, compared to boys aged 17–18 who had to use the 10 kg dumbbell, which in turn could equalize their number of repetitions and in turn reduce the power of the regression analysis to detect potential association to SOC.

Interestingly, the present study showed an inverse association between SOC and cardiovascular (CV) fitness (i.e., VO_2_max) only in boys. It seems that increased CV fitness might be a product of external stressors (e.g., high training volume and pressure), which, in turn, could reduce the coping capacity to deal with everyday stress [68]. The observed association only in boys might be connected to a higher prevalence of physical training at age 15–18, compared to girls in the present study. Specifically, sports participation on a high-level puts many demands and stress on the participants. A recently published study demonstrated that more than 50% of the athlete students frequently reported study-related stress as a predictor of general well-being and general risk of depression, while 21% showed impaired well-being [68]. In line with that, Demirel (2016) suggested reasons such as physical or somatic stress associated with vigorous exercise programs (e.g., overuse injuries) and fear of occupational careers as possible factors associated with stress for athletic students [69].

In the present study, 47% of male adolescents reported that they trained in sports at a moderate to high-intensity level at least 4 times per week, revealing their active sports participation. In accordance with that, Mayer and Thiel (2014) reported a slight, but significantly lower SOC score in elite athletes than in the general population, indicating a high demanding sports participation as a risk factor that might weaken SOC [70]. In brief, the social context of sports participation is considered to be an extremely demanding environment that includes constant competitive pressure to perform at a high level which might increase injury risk, absence from the participation and competition, cause over-training syndrome, and reduce psychological resilience to stress, which all together may lead to a decreased overall health and SOC [70,71]. 

### 4.2. Differences between Age and Sex in SOC

Generally, boys had a stronger SOC in every age group compared to girls. This is in accordance with previous studies showing boys having a stronger SOC [72,73,74]. The difference was significant at ages 15, 16, and 17, which is congruent with previous research by Moksnes et al. (2012) where boys had a significantly higher value at ages 15–16, compared to within age groups 13–14 and 17–18 [72]. The differences between the sexes in SOC in the present study might be explained by differently perceived stress in boys and girls. In brief, boys have usually reported higher levels of performance stress [73,75] and higher levels of achievement and self-relevant stressors [76], while girls intend to blame themselves in relationship problems [77] and are more affected by social relationship [78]. On the other hand, the SOC value showed small variability across age groups in both sexes, which is contrary to Antonovsky’s (1987) thesis, indicating that SOC was developing by age [12]. A possible explanation may be that adolescents between 14–18 are in the same school stage (i.e., high school) where social, emotional, and academic demands are similar across the grades.

### 4.3. Differences between Age and Sexes in Physical Activity and Fitness Level 

Expectedly, boys were taller and heavier across all age groups compared to girls. BMI showed a tendency of increasing with age. There was no difference in BMI between sexes, except in age 15, with girls having higher values. This small difference between the sexes can be explained by a low power of BMI to evaluate body composition (i.e., body fat and muscle mass). Thus, a higher BMI in girls might be a product of higher adiposity mass, while in boys it might be a result of bigger muscle mass, which is reasonable to assume when results showed higher activity level and participation rate in organized sport activities in boys than in girls. Moreover, girls had lower levels of everyday PA at age 14–17 compared to boys, which is in line with previous studies [23,79]. There were no significant differences in sedentary behavior and screen time across the age groups in either of the sexes. In general, the PA level decreased with age for both sexes, which is in line with previous studies [25]. 

### 4.4. Physical Fitness

Fitness results showed that boys had a higher cardiovascular capacity (i.e., VO_2_max) in all age groups compared to girls, which is in line with previous studies [25,80,81]. A higher PA level, cardiac size and function, as well as greater muscle mass and mechanical efficiency (e.g., larger levers) may explain the male superiority in aerobic capacity compared to female adolescents [25,82]. Additionally, boys were significantly stronger in the lower body at age 16–17, and in abdominal strength at age 15–17 compared to girls, which might explain the higher results in the cardiovascular tests too. Interestingly, the 14-year-old adolescents in the present study showed significantly higher values of VO_2_max compared to other groups. A possible explanation is that they were significantly lighter than older adolescents, which may have positively affected their relative VO_2_max. In addition, both girls and boys of this age showed to be the strongest in the abdominal and lower body tests. Moreover, they showed to have the highest everyday PA, which, in turn, could contribute to increased VO_2_max [37,83]. Results in the upper body strength were inconsistent between the sexes. However, a bias in the present study might be that boys aged 14–16 had the option of choosing either a 5 kg or a 10 kg dumbbell, compared to boys aged 17–18 who had to use the 10 kg dumbbell.

### 4.5. Strengths and Limitations

The strength of the present study was a relatively large sample size, representing the urban population of students aged 14–18 years from the middle and south of Sweden. However, the study has several limitations, which must be acknowledged. First, the cross-sectional character of the study design does not allow for the establishment of firm causal links between the variables, but only describes the relationship between them. Consequently, a longitudinal follow-up research design would be more appropriate to reveal variables that affect SOC. Second, the levels of PA, sedentary behavior and screen time were assessed by a subjective measure, including only a single question per studied measure, which could reduce the validity of the assessed construct. Third, the study did not include some potentially confounding variables (e.g., school factors, interpersonal relationships, nutritional behavior, health issues, substance use, etc.) that could affect the SOC strength. Fourth, the fact that boys aged 14–16 had the option of choosing either a 5 kg or a 10 kg dumbbell, compared to boys aged 17–18 who had to use the 10 kg dumbbell, could affect the strength score (e.g., number of repetitions), which, in turn, could reduce the power of the measure to discriminate between the age groups. Additionally, the number of 16 year old girls is quite high to other age-groups and the boys, which in turn could affect the analyses. Moreover, even though the used cardiovascular fitness tests were previously validated to estimate VO_2_max, the fact that the students could choose one of 3 different tests could affect the accuracy of the data.

## 5. Conclusions

In conclusion, even though the amount of explained variance in the regression model was low, the study revealed that adolescents with less sedentary behavior, irrespective of sex and age, had a stronger SOC and thus might have a better ability to cope with everyday stress. The results emphasize the importance of positive PA behavior in the development of a stronger SOC in adolescents. Furthermore, the findings indicate a stronger SOC and higher PA level in boys, which might affect the development of health-related strategies that target boys and girls separately. The strategies should also take into account the fact that PA consistently reduced with age. Interestingly, increased VO_2_max was associated with a weaker SOC in boys. This might be related to the reported increased training frequency, which in turn could lead to increased mental and physical burden and thus weakened SOC. This implies a need for well-designed training plans and emotional support for the participants in sport clubs that could potentially be socially vulnerable environments. 

## Figures and Tables

**Table 1:** Differences between sexes across age groups comparing anthropometric data, fitness levels, and sense of coherence.

		Age = 14	Age = 15	Age = 16	Age = 17	Age = 18	
Variables	Sex	Mean ± SD	Mean ± SD	Mean ± SD	Mean ± SD	Mean ± SD	F Test (*p* Value)
Body Mass (kg)	Girls	54.9 ± 5.4 *^¥†‡○^	61.5 ± 10.0 *	60.3 ± 9.3 *	61.2 ± 9.0 *	62.6 ± 12.3 *	4.67 (0.001)
	Boys	63.2 ± 11.4 ^¥†‡^	68.7 ± 12.8 ^¥^	69.8 ± 11.9	71.2 ± 11.4	74.6 ± 13.2	6.07 (0.001)
Body Height (m)	Girls	1.64 ± 0.03 *	1.66 ± 0.06 *	1.66 ± 0.06 *	1.65 ± 0.06 *	1.64 ± 0.08 *	1.78 (0.13)
	Boys	1.75 ± 0.08	1.78 ± 0.07	1.78 ± 0.07	1.79 ± 0.06	1.78 ± 0.07	1.23 (0.30)
BMI	Girls	20.0 ± 2.0 ^¥†○^	22.0 ± 3.3 *	21.7 ± 2.9	22.2 ± 3.0	22.9 ± 4.1	4.56 (0.00)
	Boys	20.8 ± 3.6	21.7 ± 3.4	21.8 ± 3.2	22.2 ± 3.3	23.2 ± 3.5	2.88 (0.02)
Lower body strength (seconds)	Girls	143.6 ± 103.4	113.8 ± 104.6	129.7 ± 89.2 *^¥^	122.0 ± 81.1 *	121.9 ± 75.4	2.92 (0.02)
	Boys	134.0 ± 83.1	132.7 ± 83.1	150.6 ± 102.5 ^¥^	137.7 ± 82.1	102.3 ± 74.3	4.38 (0.00)
Upper body strength (number of repetitions)	Girls	25.2 ± 13.3 *	25.7 ± 16.3	21.1 ± 14.0	25.4 ± 13.8	30.4 ± 19.1 *	1.78 (0.13)
	Boys	42.2 ± 27.6	21.7 ± 21.7	23.4 ± 67.7	22.9 ± 19.4	22.9 ± 16.2	1.64 (0.16)
Sit-ups (number of repetitions)	Girls	76.6 ± 49.0 ^¥†‡○^	52.9 ± 41.3 *	53.4 ± 31.3 *	52.6 ± 37.6 *	48.0 ± 26.9	4.61 (0.00)
	Boys	74.3 ± 48.3 ^¥^	67.5 ± 45.4 ^¥^	65.8 ± 38.2 ^¥^	66.1 ± 34.6 ^¥^	48.6 ± 13.1	3.90 (0.00)
VO_2_max (mL/kg/min)	Girls	51.1 ± 5.3 *^†‡○^	39.3 ± 9.6 *^†^	41.1 ± 8.3 *	42.7 ± 8.0 *	44.4 ± 7.2	12.85 (0.00)
	Boys	57.3 ± 8.9 ^¥†‡○^	50.7 ± 10.8 ^‡^	46.6 ± 10.7 ^†^	49.4 ± 10.9	48.6 ± 13.1	9.85 (0.001)
Sense of coherence (SOC) (total value)	Girls	54.8 ± 6.6	55.6 ± 6.7 *	55.4 ± 6.6 *	55.6 ± 7.1 *	54.7 ± 7.1	0.38 (0.82)
	Boys	56.9 ± 5.6	58.5 ± 7.3 ^¥^	56.9 ± 6.2	57.1 ± 7.0	55.5 ± 7.5	2.77 (0.03)

Legend: * Indicates significant differences between boys and girls in the accompanying age group at *p* < 0.05. ^¥^ Indicates values significantly different from those obtained in 18-year-old adolescents at *p* < 0.05. † Indicates values significantly different from those obtained in 17-year-old adolescents at *p* < 0.05. ‡ Indicates values significantly different from those obtained in 16-year-old adolescents at *p* < 0.05. ○ Indicates values significantly different from those obtained in 15-year-old adolescents at *p* < 0.05.

**Table 2:** Kruskal–Wallis and Mann–Whitney Tests comparing differences in PA between sexes and age groups. Mean value is presented for age and gender.

		Age Groups					
Variables		14	15	16	17	18	*p*-Value
Physical training (PT)	Girls	4.05	3.40 *	3.70 *	3.75	3.41	0.179
	Boys	3.89	3.92	3.92	3.81	3.47	0.550
Everyday physical activity (PA)	Girls	3.48	3.03	2.86	3.03	3.02	0.004
	Boys	3.56	3.05	2.96	3.10	2.92	0.005
Sedentary behavior (SB)	Girls	3.19	2.87	2.78 *	2.84	3.02	0.114
	Boys	2.94	2.95	2.95	2.94	2.98	0.963
Screen time (ST)	Girls	2.05 *	1.73	1.65	1.80	2.05	0.519
	Boys	1.39	1.70	1.74	1.76	1.84	0.760

Legend: Data are presented at 1–5 point scale, with higher scores indicating a higher level of PT and PA, and lower level of SB and ST; *p*-value of Kruskal–Wallis test indicates significant differences between the age groups; * Indicates significant differences between boys and girls in accompanied age group at *p* < 0.05.

**Table 3:** Factors associated with sense of coherence, separately for girls (n = 797) and boys (n = 587) (multiple linear regression analyses).

Independent Variables	Girls		Boys	
	β	*p* Value	β	*p* Value
Age	−0.033	0.357	−0.037	0.371
BMI	−0.006	0.864	0.005	0.913
Lower body strength	0.022	0.611	0.002	0.975
Upper body strength	0.041	0.307	0.046	0.321
Sit-ups	0.046	0.266	0.113	0.022 *
VO_2_max	−0.047	0.226	−0.109	0.026 *
Physical training	0.016	0.698	0.021	0.650
Everyday physical activity	−0.002	0.953	−0.029	0.517
Sedentary behavior	0.114	0.002 *	0.109	0.013 *
Screen time	0.053	0.156	0.078	0.079
Total R^2^	0.033		0.043	
Anova	F = 2.67; *p* = 0.003		F = 2.56; *p* = 0.005	

Legend: β = a standardized beta coefficient; R^2^ = the coefficient of determination. * Indicates a significant association with the criterion variable (sense of coherence).

## Data Availability

The dataset supporting the conclusion of this article is available from the authors upon reasonable request and the completion of a data transfer agreement.

## References

[1] Keyes C. (2006). Mental health in adolescence: Is America’s youth flourishing?. Am. J. Orthopsychiatry.

[2] Östenberg A.H., Pojskic H., Gilic B., Sekulic D., Alricsson M. (2022). Physical Fitness, Dietary Habits and Substance Misuse. A Cross Sectional Analysis of the Associations in 7600 Swedish Adolescents. J. Phys. Act. Health.

[3] Casey B.J., Jones R.M., Levita L., Libby V., Pattwell S.S., Ruberry E.J., Soliman F., Somerville L.H. (2010). The storm and stress of adolescence: Insights from human imaging and mouse genetics. Dev. Psychobiol. J. Int. Soc..

[4] Dvir Y., Ford J., Hill M., Frazier J. (2014). Childhood maltreatment, emotional dysregulation, and psychiatric comorbidities. Harv. Rev. Psychiatry.

[5] Kim E.Y., Miklowitz D.J., Biuckians A., Mullen K. (2007). Life stress and the course of early-onset bipolar disorder. J. Affect. Disord..

[6] Whiteford H., Degenhardt L., Rehm J., Baxter A., Ferrari A., Erskine H., Charlson F., Norman R., Flaxman A., Johns N. (2019). Global burden of disease attributable to mental and substance use disorders: Findings from the Global Burden of Disease Study. Lancet.

[7] Kieling C., Baker-Henningham H., Belfer M., Conti G., Ertem I., Omigbodun O., Rohde L., Srinath S., Ulkuer N., Rahman A. (2011). Child and adolescent mental health worldwide: Evidence for action. Lancet.

[8] Collishaw S. (2015). Annual research review: Secular trends in child and adolescent mental health. J. Child. Psychol. Psychiatry.

[9] Hofstra M., Van Der Ende J., Verhulst F. (2002). Child and adolescent problems predict DSM-IV disorders in adulthood: A 14-year follow-up of a Dutch epidemiological sample. J. Am. Acad. Child. Adolesc. Psychiatry.

[10] Geoffroy M.C., Bouchard S., Per M., Khoury B., Chartrand E., Renaud J., Turecki G., Colman I., Orri M. (2022). Prevalence of suicidal ideation and self-harm behavior children aged 12 years and under: A systematic review and meta-analysis. Lancet Psychiatry.

[11] Długosz P., Liszka D., Bastrakova A., Yuzva L. (2022). Health Problems of Students during Distance Learning in Central and Eastern Europe: A Cross-Sectional Study of Poland and Ukraine. Int. J. Environ. Res. Public Health.

[12] Antonovsky A. (1987). Unraveling the Mystery of Health: How People Manage Stress and Stay Well.

[13] Moyers S.A., Hagger M.S. (2020). Physical activity and sense of coherence: A meta-analysis. Int. Rev. Sport Exerc. Psychol..

[14] Antonovsky A. (1990). A somewhat personal odyssey in studying the stress process. Stress. Med..

[15] Lindström B., Eriksson M. (2005). Salutogenesis. J. Epidemiol. Community Health.

[16] Eriksson M., Mittelmark M.B., Mittelmark M.B., Sagy S., Eriksson M., Bauer G.F., Pelikan J.M., Lindström B., Espnes G.A. (2016). The Sense of Coherence and Its Measurement. The Handbook of Salutogenesis.

[17] Öztekin C., Tezer E. (2009). The role of sense of coherence and physical activity in positive and negative affect of Turkish adolescents. Adolescence.

[18] Linca-Ćwikła A. (2019). The sense of coherence and physical activity among secondary-school graduates living in the Lubelskie Province. Kwart. Naukowy.

[19] Moksnes U., Løhre A., Espnes G. (2013). The Association between Sense of Coherence and Life Satisfaction in Adolescents. Qual. Life Res..

[20] Biddle S.J., Ciaccioni S., Thomas G., Vergeer I. (2019). Physical activity and mental health in children and adolescents: An updated review of reviews and an analysis of causality. Psychol. Sport Exerc..

[21] Andermo S., Hallgren M., Nguyen T., Jonsson S., Petersen S., Friberg M., Romqvist A., Stubbs B., Schäfer L. (2020). School-related physical activity interventions and mental health among children: A systematic review and meta-analysis. Sports Med. Open.

[22] Rodriguez-Ayllon M., Cadenas-Sánchez C., Estévez-López F., Muñoz N., Mora-Gonzalez J., Migueles J., Molina-García P., Henriksson H., Mena-Molina A., Martínez-Vizcaíno V. (2019). Role of Physical Activity and Sedentary Behavior in the Mental Health of Preschoolers, Children and Adolescents: A Systematic Review and Meta-Analysis. Sports Med..

[23] Guthold R., Stevens G.A., Riley L.M., Bull F.C. (2020). Global trends in insufficient physical activity among adolescents: A pooled analysis of 298 population-based surveys with 1· 6 million participants. Lancet Child. Adolesc. Health.

[24] Naci H., Ioannidis J. (2013). Comparative effectiveness of exercise and drug interventions on mortality outcomes: Metaepidemiological study. Br. Med. J..

[25] Pojskic H., Eslami B. (2018). Relationship Between Obesity, Physical Activity, and Cardiorespiratory Fitness Levels in Children and Adolescents in Bosnia and Herzegovina: An Analysis of Gender Differences. Front. Physiol..

[26] Yohannes H., Östenberg A. H., Alricsson M. (2020). Health profile with body mass index and physical fitness in Swedish adolescents: A cross-sectional study. Int. J. Adolesc. Med. Health.

[27] Kriemler S., Meyer U., Martin E., van Sluijs E., Andersen L., Martin B. (2011). Effect of school-based interventions on physical activity and fitness in children and adolescents: A review of reviews and systematic update. Br. J. Sports. Med..

[28] Holt N., Deal C., Pankow K. (2020). Positive youth development through sport. Handbook of Sport Psychology.

[29] Bangsbo J., Krustrup P., Duda J., Hillman C., Andersen L.B., Weiss M., Williams C.A., Lintunen T., Green K., Hansen P.R. (2016). The Copenhagen Consensus Conference 2016: Children, youth, and physical activity in schools and during leisure time. Br. J. Sports. Med..

[30] Caspersen C.J., Powell K.E., Christenson G.M. (1985). Physical activity, exercise, and physical fitness: Definitions and distinctions for health-related research. Public Health Rep..

[31] Fritz J. (2017). Physical Activity During Growth. Effect on Bone, Muscle, Fracture Risk and Academic Performance. Ph.D. Thesis.

[32] Giroir B.P., Wright D. (2018). Physical activity guidelines for health and prosperity in the United States. J. Am. Med. Assoc..

[33] Tomkinson G., Carver K., Atkinson F., Daniell N., Lewis L., Fitzgerald J., Lang J., Ortega F. (2018). European normative values for physical fitness in children and adolescents aged 9–17 years: Results from 2 779 165 Eurofit performances representing 30 countries. Br. J. Sports. Med..

[34] Dumith S.C., Gigante D.P., Domingues M.R., Kohl H.W. (2011). 3rd Physical activity change during adolescence: A systematic review and a pooled analysis. Int. J. Epidemiol..

[35] De Looze M., Elgar F., Currie C., Kolip P., Stevens G. (2019). Gender inequality and sex differences in physical fighting, physical activity, and injury among adolescents across 36 countries. J. Adolesc. Health.

[36] Hallal P., Andersen L., Bull F., Guthold R., Haskell W., Ekelund U. (2012). Physical Activity 1 Global physical activity levels: Surveillance progress, pitfalls, and prospects. Lancet.

[37] Maddison R., Foley L., Wong H., Olds T. (2016). A great sporting nation? Sport participation in New Zealand youth. N. Z. Med. Stud. J..

[38] Campbell O.L., Bann D., Patalay P. (2021). The gender gap in adolescent mental health: A cross-national investigation of 566,829 adolescents across 73 countries. SSM Popul. Health.

[39] Hogan C., Catalino L., Mata J., Fredrickson B. (2015). Beyond Emotional Benefits: Physical Activity and Sedentary Behaviour Affect Psychosocial Resources through Emotions. Psychol. Health.

[40] Halliday A.J., Kern M.L., Turnbull D.A. (2019). Can physical activity help explain the gender gap in adolescent mental health? A cross-sectional exploration. Ment. Health Phys. Act..

[41] Flensborg-Madsen T., Ventegodt S., Merrick J. (2005). Sense of coherence and physical health. A review of previous findings. Sci. World J..

[42] Jackson A., Morrow J., Bowles H., FitzGerald S., Blair S. (2007). Construct Validity Evidence for Single-Response Items to Estimate Physical Activity Levels in Large Sample Studies. Res. Q. Exerc. Sport..

[43] Scott J., Morgan P., Plotnikoff R., Lubans D. (2015). Reliability and Validity of a Single-item Physical Activity Measure for Adolescents. J. Paediatr. Child Health.

[44] Antonovsky A. (2005). Hälsans Mysterium.

[45] Braun-Lewensohn O., Idan O., Lindström B., Margalit M. (2017). Salutogenesis: Sense of Coherence in Adolescence. The Handbook of Salutogenesis.

[46] Rivera F., García-Moya I., Moreno C., Ramos P. (2013). Developmental Contexts and Sense of Coherence in Adolescence: A Systematic Review. J. Health Psychol..

[47] Feldt T., Lintula H., Suominen S., Koskenvuo M., Vahtera J., Kivimäki M. (2007). Structural Validity and Temporal Stability of the 13-Item Sense of Coherence Scale: Prospective Evidence from the Population-based HeSSup Study. Qual. Life. Res..

[48] Cooper K. (1968). A means of assessing maximal oxygen intake: Correlation between field and treadmill testing. J. Am. Med. Assoc..

[49] Mayorga-Vega D., Bocanegra-Parrilla R., Ornelas M., Viciana J. (2016). Criterion-Related Validity of the Distance- and Time-Based Walk/Run Field Tests for Estimating Cardiorespiratory Fitness: A Systematic Review and Meta-Analysis. PLoS ONE.

[50] McSwegin P., Plowman S., Wolff G., Guttenberg G. (1998). The validity of a one-mile walk test for high school age individuals. Meas. Phys. Educ. Exerc. Sci..

[51] Åstrand P.-O., Rhymin I. (1954). A nomogram for calculation of aerobic capacity (physical fitness) from pulse rate during submaximal work. J. Appl. Physiol..

[52] Grant S., Corbett K., Amjad A., Wilson J., Aitchison T. (1995). A comparison of methods of predicting maximum oxygen uptake. Br. J. Sports Med..

[53] Thomas K., Nilsson E., Festin K., Henriksson P., Lowén M., Löf M., Kristenson M. (2020). Associations of Psychosocial Factors with Multiple Health Behaviors: A Population-Based Study of Middle-Aged Men and Women. Int. J. Environ. Res. Public Health.

[54] Hoare E., Milton K., Foster C., Allender S. (2016). The Associations between Sedentary Behaviour and Mental Health among Adolescents: A Systematic Review. Int. J. Behav. Nutr. Phys. Act..

[55] Liu M., Wu L., Yao S. (2016). Dose-response Association of Screen Time-based Sedentary Behaviour in Children and Adolescents and Depression: A Meta-analysis of Observational Studies. Br. J. Sport. Med..

[56] Iannotti R., Kogan M., Janssen I., Boyce W. (2009). Patterns of Adolescent Physical Activity, Screen-Based Media Use, and Positive and Negative Health Indicators in the U.S. and Canada. J. Adolesc. Health.

[57] Babic M., Smith J., Morgan P., Eather N., Plotnikoff R., Lubans D. (2017). Longitudinal Associations between Changes in Screen-time and Mental Health Outcomes in Adolescents. Ment. Health Phys. Act..

[58] Vuong A.T., Jarman H.K., Doley J.R., McLean S.A. (2021). Social media use and body dissatisfaction in adolescents: The moderating role of thin-and muscular-ideal internalisation. Int. J. Environ. Res. Public Health.

[59] Suchert V., Hanewinkel R., Isensee B. (2015). Sedentary Behavior and Indicators of Mental Health in School-aged Children and Adolescents: A Systematic Review. Prev. Med..

[60] Asare M., Danquah S. (2015). The Relationship between Physical Activity, Sedentary Behaviour and Mental Health in Ghanaian Adolescents. Child Adolesc. Psychiat. Ment. Health.

[61] Luppino F., de Wit L., Bouvy P., Stijnen T., Cuijpers P., Penninx B., Zitman F. (2010). Overweight, obesity, and depression: A systematic review and meta-analysis of longitudinal studies. Arch. Gen. Psychiatry.

[62] Åvitsland A., Leibinger E., Haugen T., Lerum Ø., Solberg R., Kolle E., Dyrstad S. (2020). The Association between Physical Fitness and Mental Health in Norwegian Adolescents. BMC Public Health.

[63] Janssen A., Leahy A., Diallo T., Smith J., Kennedy S., Eather N., Mavilidi M., Wagemakers A., Babic M., Lubans D. (2020). Cardiorespiratory Fitness, Muscular Fitness and Mental Health in Older Adolescents: A Multi-level Cross-sectional Analysis. Prev. Med..

[64] Smith J., Eather N., Morgan P., Plotnikoff R., Faigenbaum A., Lubans D. (2014). The Health Benefits of Muscular Fitness for Children and Adolescents: A Systematic Review and Meta-Analysis. Sports Med..

[65] Lubans D.R., Cliff D.P. (2011). Muscular fitness, body composition and physical self-perception in adolescents. J. Sci. Med. Sport.

[66] Padilla-Moledo C., Ruiz J., Ortega F., Mora J., Castro-Piñero J. (2012). Associations of Muscular Fitness with Psychological Positive Health, Health Complaints, and Health Risk Behaviors in Spanish Children and Adolescents. J. Strength Cond. Res..

[67] Volaklis K., Mamadjanov T., Meisinger C., Linseisen J. (2019). Association between Muscular Strength and Depressive Symptoms. Wien. Klin. Wochenschr..

[68] Bastemeyer C., Kleinert J. (2021). Mental Health in Sports Students—A Cohort Study on Study-related Stress, General Well-being, and General Risk for Depression. J. Phys. Educ. Sport..

[69] Demirel H. (2016). Have University Sport Students Higher Scores Depression, Anxiety and Psychological Stress?. Int. J. Environ. Sci. Educ..

[70] Mayer J., Thiel A. (2014). Health in Elite Sports from a Salutogenetic Perspective: Athletes’ Sense of Coherence. PLoS ONE.

[71] McAllister D.R., Motamedi A.R., Hame S.L., Shapiro M.S., Dorey F.J. (2001). Quality of Life Assessment in Elite Collegiate Athletes. Am. J. Sports Med..

[72] Moksnes U., Espnes G., Lillefjell M. (2012). Sense of coherence and emotional health in adolescents. J. Adolesc..

[73] Moksnes U., Espnes G., Haugan G. (2014). Stress, Sense of Coherence and Emotional Symptoms in Adolescents. Psychol. Health.

[74] Würtz E., Fonager K., Tølbøll Mortensen J. (2015). Association between Sense of Coherence in Adolescence and Social Benefits Later in Life: A 12-year Follow-up Study. BMJ Open.

[75] Lavoie L., Dupéré V., Dion E., Crosnoe R., Lacourse E., Archambault I. (2019). Gender Differences in Adolescents’ Exposure to Stressful Life Events and Differential Links to Impaired School Functioning. J. Abnorm. Child Psychol..

[76] Rudolph K., Hammen C. (1999). Age and gender as determinants of stress exposure, generation, and reactions in youngsters: A transactional perspective. Child Develop..

[77] Rudolph K. (2002). Gender differences in emotional responses to interpersonal stress during adolescence. J. Adolesc. Health.

[78] Rose A. (2002). Co–rumination in the friendships of girls and boys. Child Dev..

[79] Gomes T.N., Hedeker D., dos Santos F.K., Souza M., Santos D., Pereira S., Katzmarzyk P.T., Maia J. (2017). Relationship between sedentariness and moderate-to-vigorous physical activity in youth: A multivariate multilevel study. Int. J. Environ. Res. Public Health.

[80] Dencker M., Wollmer P., Karlsson M.K., Lindén C., Andersen L.B., Thorsson O. (2012). Body fat, abdominal fat and body fat distribution related to cardiovascular risk factors in prepubertal children. Acta Paediatr..

[81] Tuan S., Su H., Chen Y., Li M., Tsai Y., Yang C., Lin K. (2018). Fat mass index and body mass index affect peak metabolic equivalent negatively during exercise test among children and adolescents in Taiwan. Int. J. Environ. Res. Public Health.

[82] Manna I. (2014). Growth development and maturity in children and adolescent: Relation to sports and physical activity. Am. J. Sports Sci. Med..

[83] Eime R., Harvey J., Charity M., Casey M., Westerbeek H., Payne W. (2016). Age profiles of sport participants. BMC Sports Sci. Med. Rehabil..

